# GREATER HYPOGLYCEMIA UNAWARENESS IN OLDER COMPARED TO YOUNGER RURAL VETERANS WITH TYPE 2 DIABETES

**DOI:** 10.1093/geroni/igz038.1726

**Published:** 2019-11-08

**Authors:** Willy Marcos Valencia, Kaicheng Wang, Kiranmayee Muralidhar, Stuti Dang

**Affiliations:** 1 Miami Veterans Affairs Healthcare System- GRECC, Miami, Florida, United States; 2 Department of Public Health Sciences, University of Miami Miller School of Medicine, Miami, Florida, United States; 3 Miami VA GRECC, Miami, Florida, United States

## Abstract

Hypoglycemia is of great concern in older patients, especially when complicated with multimorbidity and geriatric syndromes. We implemented a telemedicine model to address hypoglycemia knowledge, risk factors, incidence and comanagement with their primary care teams (PCT). We identified 166 consecutive rural veterans with high hypoglycemia risk, based on a local medication database (sulfonylureas and insulin), age, and recent glycated hemoglobin A1c (HbA1c). We conducted a telephone medication reconciliation and survey assessing glucose self-monitoring (GSM), hypoglycemia knowledge and symptoms. Variables were tested using chi-square, Fisher’s, and one-way ANOVA. Multivariable logistic regression model was built to assess the association of hypoglycemia and age group, adjusted with treatment, HbA1c%, self-monitoring, and knowledge. There were 54 veterans aged <65 (younger), and 112 veterans aged ≥65 years (older). Average HbA1c was higher in younger than older (8.20±1.96 vs 7.43±1.34%, p=.003). There was no difference in treatment regimens, but the older had greater GSM (p=.028) and lower hypoglycemia symptom knowledge (p=.026). Symptomatic hypoglycemia was greater in younger versus older (50.0% vs 30.4%, p=0.014). Recent (past-2-weeks) hypoglycemic events were more frequent in younger than older (24.1 vs 1.79%, p<.001). Regression analyses showed that younger veterans were more likely to have hypoglycemia (OD=2.37, 95% CI 1.11-5.04). Our results indicate a great need to evaluate older adults with high hypoglycemia risk, in whom we observed less reports of hypoglycemia albeit with similar regimens and lower HbA1c. We suspect greater hypoglycemia unawareness, thus we are implementing a project using continuous glucose monitoring in this high-risk population.

